# Standardization of Manual Method of Immunohistochemical Staining for Breast Cancer Biomarkers at Tertiary Cancer Care Center: An Audit

**DOI:** 10.7759/cureus.25773

**Published:** 2022-06-08

**Authors:** Manjit K Rana, Amrit Pal S Rana, Aklank Jain, Akhilesh Pathak, Utkarshni Khera, Uttam Sharma, Akriti Jindal, Karuna Singh

**Affiliations:** 1 Pathology and Laboratory Medicine, All India Institute of Medical Sciences, Bathinda, IND; 2 General Surgery, All India Institute of Medical Sciences, Bathinda, IND; 3 Zoology, Central University of Punjab, Bathinda, IND; 4 Forensic Medicine, All India Institute of Medical Sciences, Bathinda, IND; 5 Pathology/Lab Medicine, All India Institute of Medical Sciences, Bathinda, IND; 6 Radiation Oncology, Advanced Cancer Institute, Baba Farid University of Health Sciences Faridkot, Bathinda, IND

**Keywords:** manual method, formalin, preanalytic factors, antigen retrieval, immunohistochemistry

## Abstract

Immunohistochemistry (IHC) is a necessary ancillary technique in surgical pathology laboratories, particularly for oncology tissue specimens. Automation in the IHC technique has an advantage over manual methods in terms of quality, except for the cost of the equipment. Thus, the manual method of IHC staining is the preferred method of choice in countries with limited resources. However, standardization of all steps in the preanalytic phase is critical to obtain reliable immunohistochemistry test results. The current audit was conducted to describe the preanalytic factors affecting manual IHC methods. The most important preanalytic factors were fixative, the composition of dehydrate, pH, drying of sections, and heat-mediated antigen retrieval method (HMAR). The domestic pressure cooker method was found to be the best for HMAR.

## Introduction

In an era of surgical oncologic histopathology, increasing attention is paid to immunohistochemistry (IHC) in pathology laboratories. Automated IHC techniques have been adopted worldwide; however, their automation is cost-prohibitive in low-income countries [1]. Manual IHC methods require careful consideration of preanalytic factors to maintain a high quality of routine work. Many laboratories scrutinize the results related to IHC stain quality [2]. From a practical point of view, one of the most difficult issues in the standardization of IHC is the effect of formalin on antigenicity. Another important factor is the variation in the fixation/processing procedures. The retrieval of antigen from tissue sections is the main affected area. We present our experience with a manual method of staining for estrogen receptor (ER), progesterone receptor (PR), and receptor tyrosine-protein kinase erbB-2 (HER2/neu) in breast carcinoma. This study aimed to audit the quality of IHC staining in our institution.

## Materials and methods

We used cervical tissue to standardize breast carcinoma biomarkers (i.e., ER and PR) and carcinoma breast tissue for HER2/neu. Tissues segments from 50 breast tumors were taken and divided into two groups: group A and group B. Tissues of group A were fixed in 10% formalin, and group B were fixed in 10% neutral phosphate-buffered formalin (NBF) for a minimum of eight hours and maximum for 72 hours. The tissues of both the groups were further stored in cassettes for six to 12 hours in the same fixative, followed by processing. Group A tissues were fixed in isopropyl alcohol, and group B tissues were fixed in ethyl alcohol separately for tissue dehydration during embedding in paraffin and for dehydration of the stained sections. IHC was performed manually on both breast tissue groups. Representative sections from paraffin-embedded blocks were cut to 2-3 μm on coated (polylysine) glass slides. Tissue sections were fixed in precooled 10% NBF for 10 minutes at room temperature and washed with phosphate-buffered saline (pH 7.4). The sections were washed in deionized water before use and then dipped in a peroxide block for 10 minutes, followed by washing in Tris-buffered solution (TBS) for five minutes. The protocol recommended by the supplier was used for the steps. Antigen retrieval solution, peroxide block, power block, primary antibody, super-enhancer, poly-horseradish peroxide (poly-HRP), and substrate solution provided by the manufacturer (Biogenex, Fremont, US) were used. However, different antigen retrieval methods were applied, including a hot air oven, pressure cooker, water bath, and microwave (temperature was adjusted according to the manufacturer's instructions, Biogenex). The sections were dipped in the primary antibody for one hour, washed with TBS, and then dipped in HRP polymer for 30 minutes. The sections were dipped in 3, 3'-diaminobenzidine tetrahydrochloride chromogen for five minutes and then in hematoxylin for five minutes. All slides were evaluated for at least 10 high-power fields for each tumor section. The rate and intensity of nuclear staining were calculated for ER and PR receptors, and complete membranous staining was calculated for HER2/neu. To avoid potential variations among different batches of IHC staining procedures, all sections using a different antigen retrieval method were stained in a single batch for a more accurate comparison.

## Results

**Figure 1:**
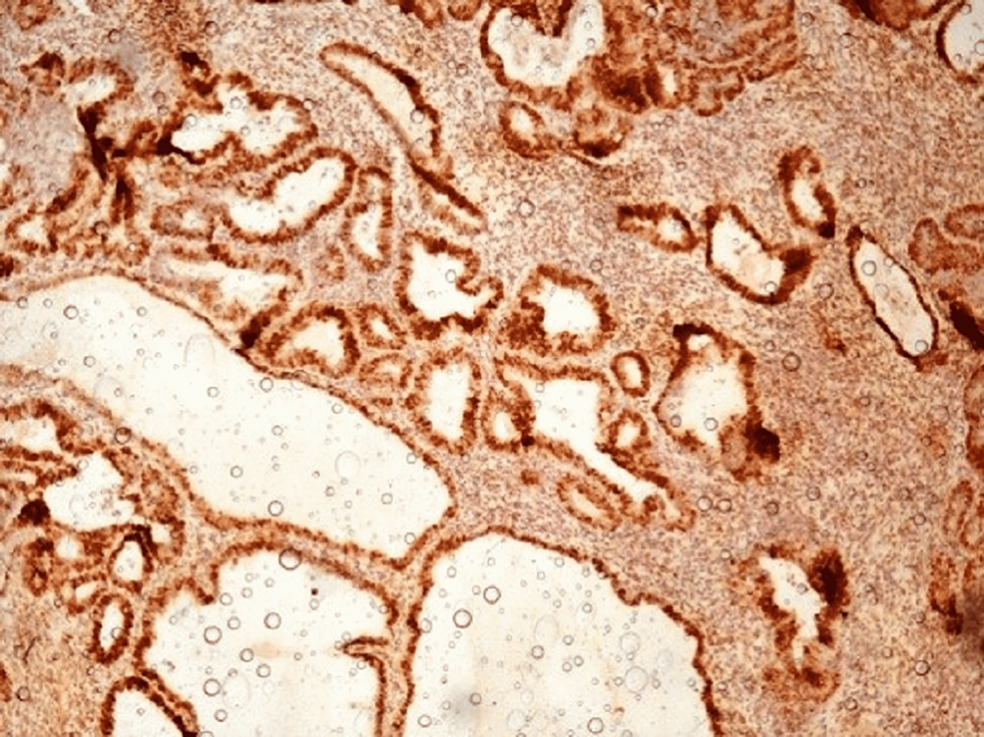
Background staining (cervical tissue immunoreactive for ER antibody used as control; 10X) ER - estrogen receptor

**Figure 2:**
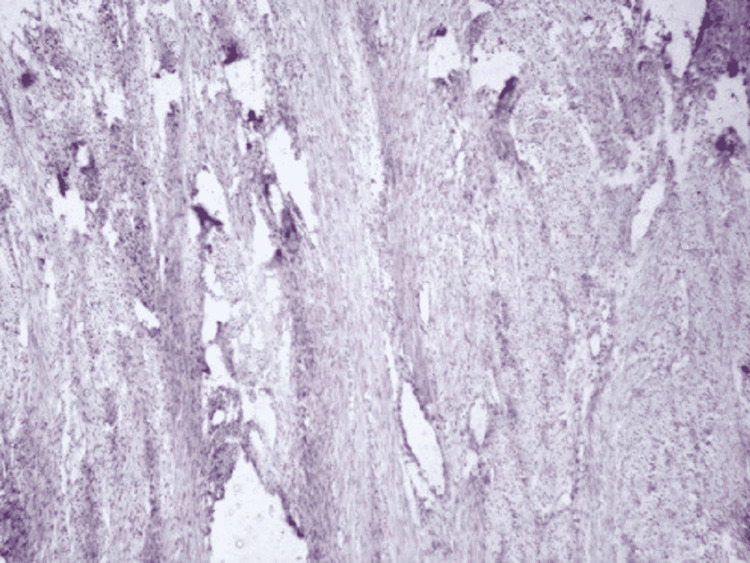
Tissue damage (endometrial tissue non-immunoreactive for ER antibody used as control; 10X) ER - estrogen receptor

**Figure 3:**
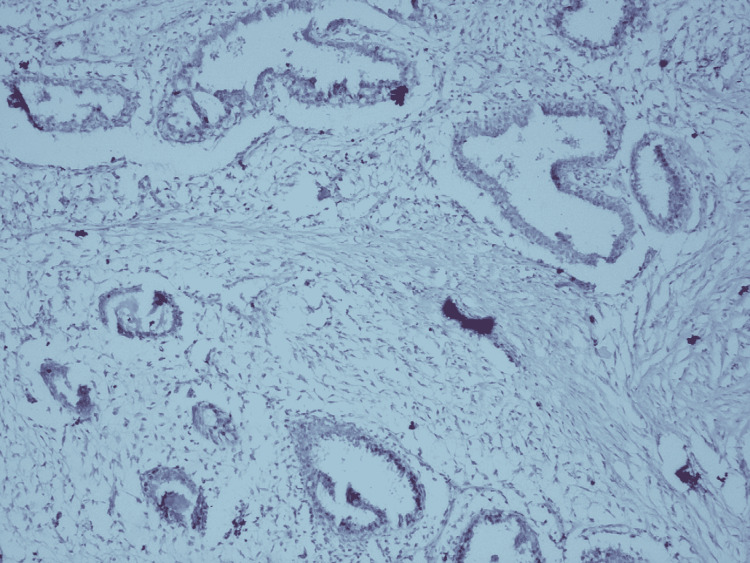
Cervical tissue immunoreactive for ER antibody used as control; 10X ER - estrogen receptor

The primary challenge was tissue floating in group A tissues fixed in 10% formalin and dehydrated in isopropyl alcohol. Whereas in group B tissues, no tissue floatation was seen. The tissue floatation was seen corrected by maintaining quality checks using 10% NBF for tissue fixation and processing quality dehydrates such as ethyl alcohol while performing manual IHC staining. Antigen retrieval presented major challenges (Table 1). The antigen retrieval done keeping slides in antigen retrieval solution in a hot air oven at 160 degrees C for one hour showed background staining and tissue floatation (Figure 1). Antigen retrieval done with a microwave also showed tissue floatation, uneven AR, tissue damage, and edge artifact (Figure 2). No antigen could be retrieved with the use of a water bath. The best results were obtained by using a domestic pressure cooker with special attention given to the number of whistles required for the retrieval of antigens for membrane as well as nuclear markers (Figure 3). 

**Table 1:** Antigen retrieval results AR - antigen retrieval; HER2/neu - receptor tyrosine-protein kinase erbB-2 *Figure 1, **Figure 2, ***Figure 3

Method of retrieval	Temperature	Time	AR solution used/not used	AR	Demerits
Hot air oven	160°C	One hour	Used	Yes	Background staining*, tissue floating
Microwave	Medium	Five to eight minutes	Used	Yes	Tissue floating, uneven AR, tissue damage, edge artifact**
Water bath	As recommended	As recommended	Used	No	
Pressure cooker	One whistle for HER2/neu; three whistles for the rest of the markers	-	Used	Yes***	

## Discussion

Formaldehyde in the formalin reacts with tissue proteins to create hydroxymethyl groups, which cross-react with other proteins to form crosslinks. Tissue remains well preserved due to crosslinks; however, these cause steric interference with antibody binding, affecting immunostaining. Antigen retrieval is a procedure that reveals antigen immunoreactivity [3]. IHC is a reliable ancillary technique to provide additional information about the typing and sub-typing of various malignancies. However, due to the presence of variable factors affecting the IHC test results and the lack of standardization of IHC laboratories, inconsistent IHC assay results are frequently reported [4-6]. The standardization of IHC is a big challenge for pathology laboratories and requires significant effort [7]. Libard et al. observed that IHC being an unpredictable technique, needed a detailed methodological description [8]. Adequately fixed and processed paraffin-embedded tissue is an important factor affecting the outcome and reliability of IHC techniques. Tissue was fixed in 10% NBF for a minimum of eight hours in a tissue container with the required amount of fixative, and tissue was fixed in formalin for six to 12 hours before tissue processing. Isopropyl alcohol used in different grades (50%, 70%, 90%, 100%) is a good and cost-effective alternative to ethyl alcohol in tissue processing. In our experience, tissue floating was observed using isopropyl alcohol; therefore, its use should be discouraged in manual IHC staining [9,10]. During the process, the tissue undergoes fixation and dehydration, and ethyl alcohol used in different grades (50%, 70%, 90%, 100%) is found to accurate fixative as well as dehydration solution, so no tissue floatation was seen [11].

Manual heat-mediated antigen retrieval is the most challenging task, and various methods have been used. A hot air oven is also a good method, but there are some drawbacks in the form of tissue floating at high temperatures, poor quality of staining, even folding in some cases, and damage or detachment of tissues from the slides. However, Vinod et al. recommended that the tissue on the slide should be covered with a plain slide to provide good results [12]. Another heating device used was a microwave oven, and challenges remained in tissue floating, folding, uneven antigen retrieval, and edge artifacts. Other limitations include the restricted use of metal trays for microwaves. No antigen was retrieved using a water bath [13]. A domestic pressure cooker was the best method for manual IHC staining. We took the precaution to adjust the duration and pressure for antigen retrieval by counting the number of whistles emitted by the cooker: one whistle for standardized HER2 antigen and three for the remaining IHC markers. The domestic pressure cooker was cost and time effective [14] and offered other benefits, including the ability to process large batches of slides and use metal slide racks. Drying should be done carefully, as IHC technicians can easily overlook it. The sections were not dried at any step of the manual IHC staining method. Drying sections during staining can result in increased nonspecific background staining. Engel et al. observed that drying at 60°C for four hours reduced immunostaining intensity in 23% of target antigens. Also, 69% of the target antigens may show decreased staining intensity and increased nonspecific background staining after incubating the sections at 70°C for eight hours [15].

As tumor biomarkers are important for the management of carcinoma and treatment therapy is dependent on the immunohistochemical expression of the cancer biomarker, so IHC technique used should be appropriate [16]. Krenacs et al. and Kim et al. also studied various antigen retrieval methods and emphasized that the most important factors affecting the IHC techniques are pre-analytic factors and should be taken care of [13,17]. 

The limitation of our study is that the manual heat-mediated antigen retrieval method is more compatible with laboratories with a lesser workload.

## Conclusions

Our goal was to audit the quality of the manual method of IHC staining in our institution and find the easiest and most cost-effective technique. For adequate standardization of manual IHC technique, more emphasis should be given to pre-analytic factors. One of the most challenging issues was formalin's adverse influence on antigenicity, background staining, and the effect of alcohol type used for tissue processing on tissue floatation. The gold standard fixative used for correction of the background staining was 10% NBF instead of 10% formalin. The absolute alcohol was found to be a good alcohol-based solution used to overcome shortcomings of tissue floatation as compared to isopropyl alcohol. Antigen retrieval is a crucial step in the manual method of IHC, and the domestic pressure cooker method was found to be the best and most cost-effective method for antigen retrieval. 
